# Ag nanoparticles immobilized on new magnetic alginate halloysite as a recoverable catalyst for reduction of nitroaromatics in aqueous media

**DOI:** 10.1038/s41598-021-96421-5

**Published:** 2021-08-24

**Authors:** Pourya Mohammadi, Majid Heravi, Mansoureh Daraie

**Affiliations:** Department of Chemistry, Faculty of Physics and Chemistry, Alzahra University, Vanak, PO Box 1993891176, Tehran, Iran

**Keywords:** Chemistry, Catalysis, Catalyst synthesis

## Abstract

Amines can be applied in the synthesis of various important compounds such as dyes, drugs, polymers, pharmaceutical products, and biologically active materials. The significant subject in the preparation of amines is the selection of the most effective heterogeneous catalyst to get the best catalytic efficiency, stability, recoverability, and reusability. For this target, we prepared new alginate magnetically recoverable nanocatalyst by stabilization of Ag nanoparticles on the surface of the halloysite (HS) [HS-Alginate-Ag/Fe_3_O_4_]. Several detection methods confirmed the production of HS-Alginate-Ag/Fe_3_O_4_ nanocatalyst and the results obtained were well explained in the context. HS-Alginate-Ag/Fe_3_O_4_ presented good catalytic performance for the hydrogenation of nitro compounds using NaBH_4_ as the reducing agent and hydrogen donor. The good activity and durability of this catalyst can be attributed to the good dispersion and nano-sized particle of silver nanoparticles.

## Introduction

The selective hydrogenation of nitro compounds to the corresponding amino compounds is one of the main synthetic processes from the industrial and academic fields because the obtained amines are commercially valuable starting materials and adaptable intermediates in the production of pharmaceuticals, rubber auxiliaries, dyestuffs, explosives, antioxidants, pesticides, agrochemicals, spices, polymers, and drugs^1–4^ while nitro compounds are the carcinogenic contaminants and toxic bio-refractories entered in industrial and agricultural wastewater^5^. Therefore, finding effective methods to eliminate these pollutants is essential. In this regard, various reduction systems and catalysts have been applied for the reduction of nitro compounds^6–8^. The classic method of synthesizing amine compounds is to reduce nitro compounds by metallic reagents, which resulting in considerable pollution in the environment^9^. However, this method has various disadvantages, such as simple corrosion of equipment, environmentally hazardous, low performance, and difficulties in continuous products and their separation^10^. Hence, synthetic processes including cheaper and clearer alternatives are required. There are several methods for the reduction of nitro compounds including chemical reduction, electro-reduction, and chemical hydrogenation^11^. Among them, the catalytic hydrogenation of these compounds has received special attention owing to its excellent performance and selectivity. One of these methods is the use of NaBH_4_ in water as a hydride source. However, reducing nitro groups with NaBH_4_ in the lack of catalysts is very time-consuming and tedious, so various metal catalysts have been utilized to perform this reduction^12–14^.

Recently, metal-based nanoparticles synthesized via the green process without any stabilization agent are broadly employed in heterogeneous and homogeneous catalysis systems because of their particular structures and characteristics^4,15^. The use of metal nanoparticles as the nanocatalyst in the presence of NaBH_4_ as a hydride donor is an eco-friendly approach for the hydrogenation of nitro compounds because this reaction can be done under moderate conditions without acid sewage^16^. Among metallic nanoparticles, silver nanoparticles have interested very consideration due to their good catalytic activities, extremely large surface area, excellent conductivity, and high stability^17,18^. Such catalytic systems provide highly effective and selective organic reactions. Although Ag nanoparticles as catalysts have various benefits, great cost, aggregation, and problems in recovery are the apparent difficulties to be faced. Nowadays, an improved approach is to deposit these nanoparticles on appropriate supports^19^. Since some supports are costly for practical application, hence, finding affordable and accessible support is necessary.

Halloysite (HS) has the potential to be used as a catalyst carrier to inhibit the aggregation of silver nanoparticles. HS with a formula Al_2_Si_2_O_5_(OH)_4_·nH_2_O (n = 0, 2) is naturally existing in soils and weathered rocks^20,21^. It is a clay mineral having a hollow tubular structure with a multi-layer wall in the nanometer range (lengths of 300 ~ 900 nm, inside diameters of 10 ~ 25 nm, and outside diameters of 50 ~ 100 nm) and a high specific surface area^22,23^. As well as HS owns many reactive groups on inside and outside surfaces^24–26^. In comparison with other tubular nanostructures e.g. carbon nanotubes, HS is eco-friendly, biocompatible, and cost-effective. Due to its high specific surface area and hollow structure, HS can serve as promising support for various applications^27–30^. HS has been investigated as high-efficiency support for various nanoparticle such as Pd^31^, ZnO^32^, TiO_2_^33^, Au^34^ resulting in effective catalyzes with adjustable properties.

In recent years, polysaccharides including chitosan, gelatin, and alginate are investigated as a most appropriate substrate for metal nanoparticles owing to their biocompatible, biodegradable, rheological, and non-toxic features^35–38^. Among them, alginate is a non-toxic and anionic compound, naturally exiting in bacteria and brown algae. Its polysaccharide chain contains β-d-mannuronate (M)and α-l-guluronate (G), linearly linked by 1,4-glycosidic units^39^. Moreover, there are abundant of hydroxyl and carboxyl groups in this macromolecule which can be suitably connected to other compounds.

Over the last years, magnetic nanoparticles (e.g. Fe_3_O_4_) have been broadly studied as a suitable compound for the separation of compounds in numerous biological and industrial fields^40^. Their magnetic property are them effectively and readily separated from the reaction media by using an external magnet.

In this study, we describe the synthesis of Ag nanoparticles stabilized on new alginate magnetic halloysite as a magnetically separable nanocatalyst and it was also investigated for its catalytic activity in hydrogenation of nitro aromatic compounds with NaBH_4_ as a mild hydride donor. It is found that the as-synthesized HS-Alginate-Ag/Fe_3_O_4_ can be used as a new, reusable and efficient nanocatalyst for the reduction of nitro aromatic compounds into the target amine derivatives. To the best of our knowledge, HS-Alginate-Ag/Fe_3_O_4_ has not been reported as the nanocatalyst for the reduction of nitro aromatic compounds so far.

## Experimental

### Materials and instruments

All the chemical material and solvents such as acetonitrile (CH_3_CN, 99%), ethanol (CH_3_CH_2_OH, 96%), dichloromethane (CH_2_Cl_2_, 99%), N, N-Dimethylformamid (HCON (CH_3_)_2_, DMF, 99.8%), and ammonia solution (NH_4_OH, 25%) were used with high purity from Merck. The required chemicals such as iron (III) chloride and iron (II) (FeCl_3_.6H_2_O, 97% and FeCl_2_.4H_2_O, 99%), Sodium borohydride (NaBH_4_, 98%), silver nitrate (AgNO_3_, 99%), halloysite, alginate, and nitroaromatic derivations were prepared from Sigma-Aldrich.

For determining the crystalline and phase structure of the synthesized nanocomposite, Transmission electron microscopy (TEM) imaging was performed with a CM30, Philips, Germany operating at 300 kV. Field emission scanning electron microscopy (FESEM) images equipped with an EDS attachment were taken on VEGA3, Tescan, USA. X-ray diffraction patterns were obtained on a PW 1800 X-ray diffractometer (Philips, Netherlands) with Cu Kα radiation (l = 0.154056 nm). Fourier transform infrared (FTIR) spectra were recorded using an FTIR apparatus (SHIMADZU, Japan) and in the range of 4000–400 cm^−1^.

### Synthesis of HS-Alginate-Ag/Fe_3_O_4_ nanocatalyst

**Figure 1:**
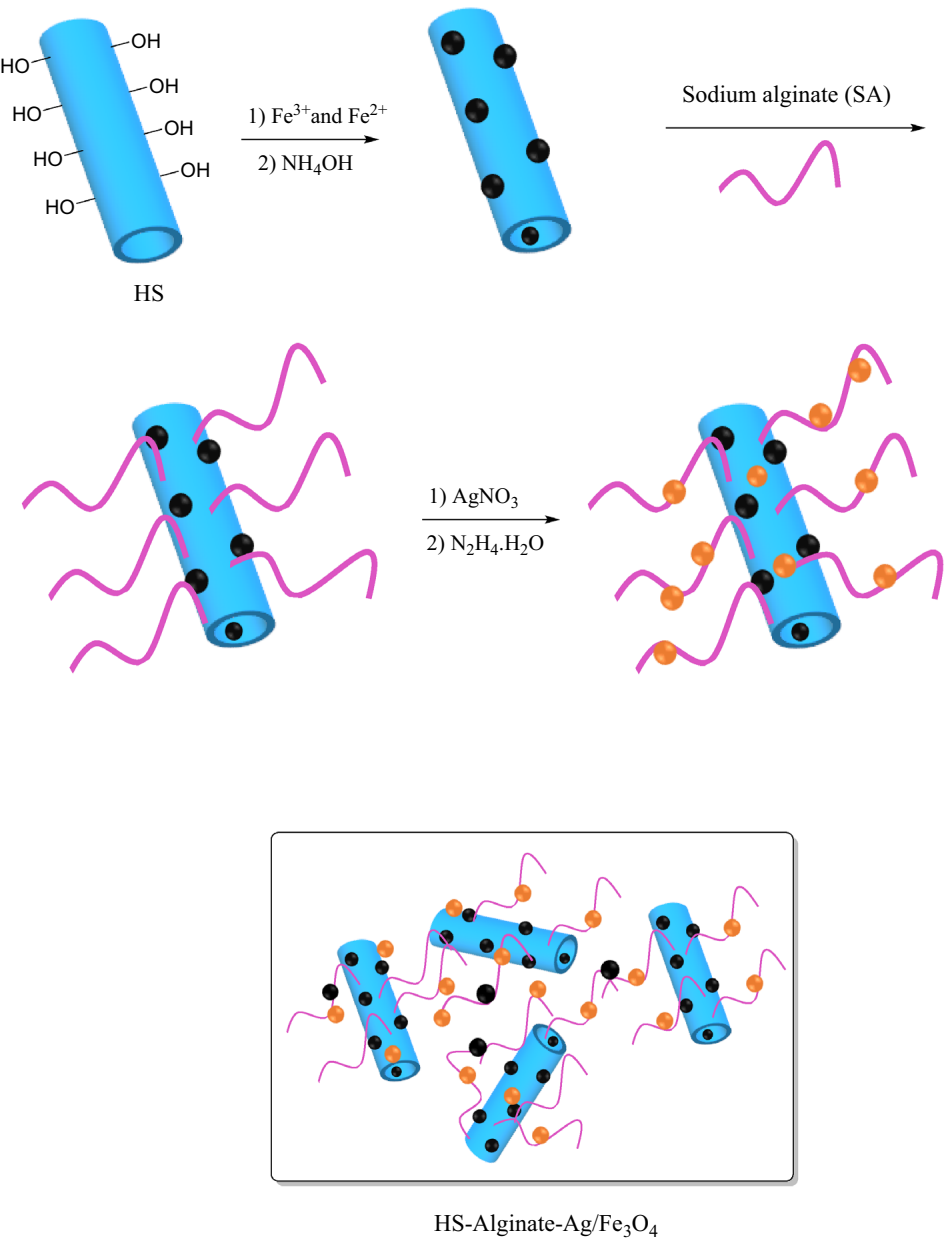
The nanocatalyst synthesis pathway (drawn with ChemDraw Pro 12.0 software (https://en.freedownloadmanager.org/Windows-PC/ChemDraw-Pro.html)).

The synthesis of HS-Alginate-Ag/Fe_3_O_4_ nanocomposites involves several steps as follows (Fig. 1):

- (a) Synthesis of Hs-Fe_3_O_4_: The 2.5 g of the HS was dispersed in 120 mL of deionized water for 15 min. Then, iron (III) chloride (1.37 g) and iron (II) (0.5 g) were added to the above suspension. This suspension was stirred at 70 °C under an N_2_ atmosphere. Then, 11 mL of concentrated ammonia was added to the above mixture and stirred for one hour. At the completion of the reaction, the product by an external magnet was separated, washed 3 times with distillated water, and dried at room temperature.
- (b) Synthesis of HS-Alginate/Fe_3_O_4_: In this stage, 500 mg of halloysite/Fe_3_O_4_ in 60 mL was dispersed in water for 20 min. Then 100 mg of alginate was added to the above solution and stirred for 5 h. Finally, the precipitation by using an external magnet was separated, washed 3 times with distillated water, and dried at room temperature.
- (c) Synthesis of HS-Alginate-Ag/Fe_3_O_4_: At first, 500 mg of HS-Alginate/Fe_3_O_4_ in 50 mL of water was stirred for 90 min at 25 °C, and then 1.4 mmol AgNO_3_ was added to the above mixture. In the next step, 0.25 mL of hydrazine hydrate in 1 mL of distilled water was added dropwise to the above solution and stirred at 25 °C for overnight. Subsequently, the precipitation by a magnet was isolated and washed three times with distilled water, and dried completely.

### The procedure for reduction of nitro aromatic compounds

To a round bottomed flask containing nitro compound (1 mmol) and 5 mL H_2_O, 0.03 g catalyst was added and the mixture was vigorously stirred at room temperature. Then NaBH_4_ was added to the suspension and the reaction temperature was raised to 50 °C. After completion of the reaction (monitored by TLC), the catalyst by an external magnetic was separated and then the precipitate was recrystallized from EtOH to give pure products (Table 2).

## Result and discussion

### Catalyst formation verification

**Figure 2:**
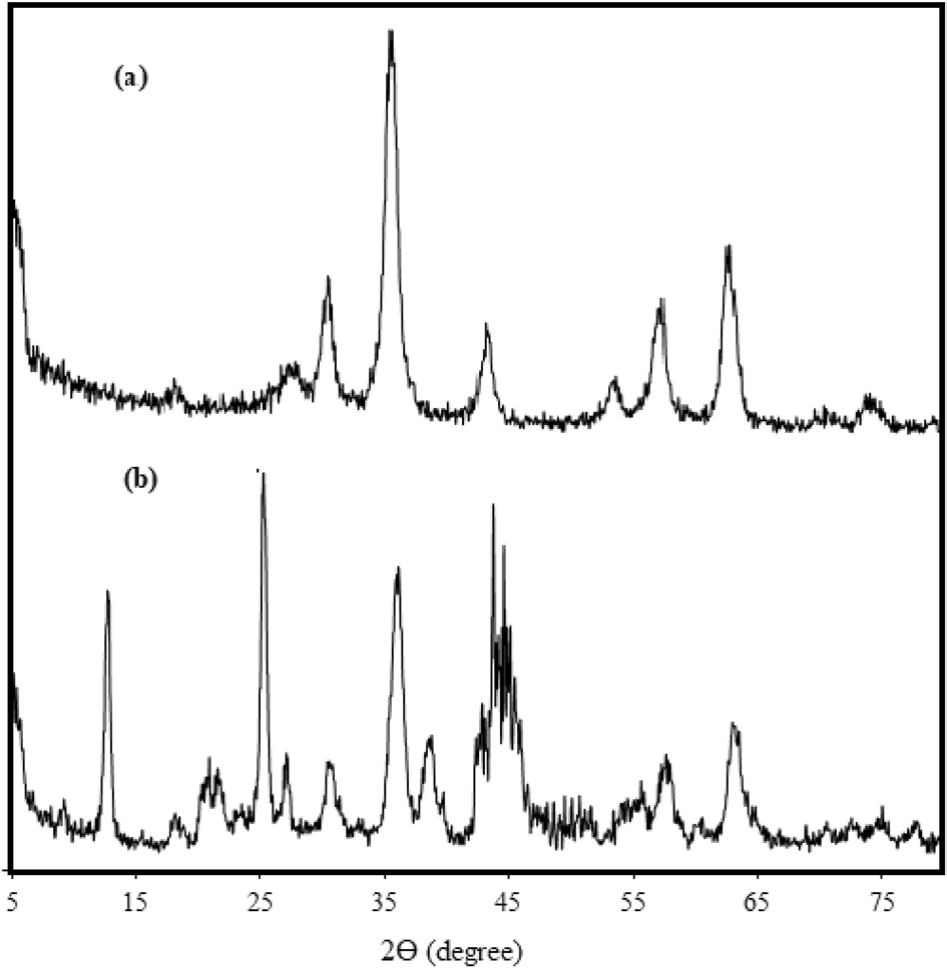
XRD patterns of (**a**) Fe3O4, and (**b**) HS-Alginate-Ag/Fe_3_O_4_ nanocomposite.

XRD analyses authenticate the Fe_3_O_4_ nanoparticles and HS-Alginate-Ag/Fe_3_O_4_ nanocomposite as presented in Fig. 2. As shown in Fig. 2a, the diffraction peaks at 30.79°, 36.14°, 44.09°, 57.89°, and 63.04° corresponding to the Fe_3_O_4_ nanoparticles interlayer spacing (220), (311), (400), (511), and (440) reflection plane, respectively. In Fig. 2b, in addition to peaks related to Fe3O4 nanoparticles, the diffraction peaks at 2θ = 12.74°, 20.84°, 25.24°, and 55.84° correspond to the HS interlayer spacing (001), (110), (002), and (114) reflection plane, respectively. In addition, the diffraction peaks at 38.54°, 44.09°, 63.04°, and 78.09°, correspond to the Ag nanoparticles interlayer spacing (111), (200), (220), and (311) reflection plane, respectively.

**Figure 3:**
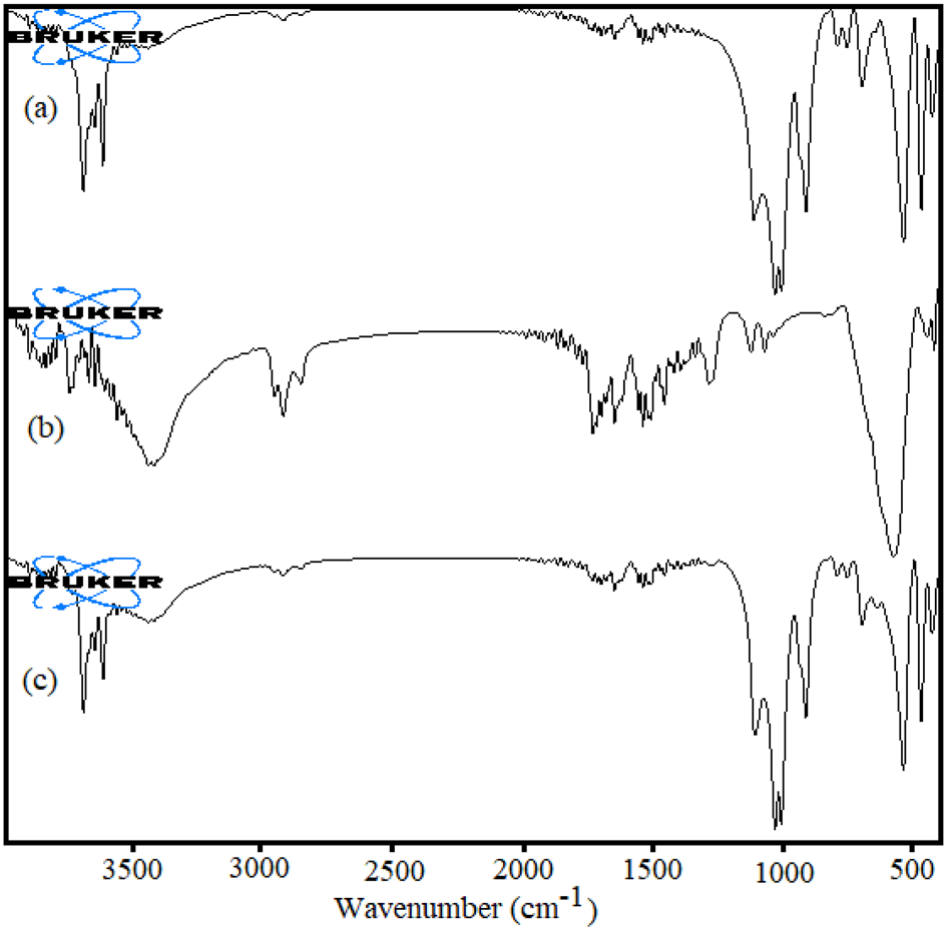
FTIR spectra of (**a**) HS-Fe_3_O_4_, (**b**) HS-Alginate /Fe_3_O_4_, and (**c**) HS-Alginate-Ag/Fe_3_O_4_.

FTIR spectra of HS /Fe_3_O_4_, HS-Alginate/Fe_3_O_4_, and HS-Alginate-Ag/Fe_3_O_4_ were recorded, Fig. 3. In the FTIR spectrum of HS/Fe_3_O_4_ (Fig. 3a), the typical adsorption peaks of Si–O stretching located at 1091 cm^−1^. The vibrations at 534 cm^−1^ are assigned to the Al–O–Si group. In addition, the absorption bands at 3695 cm^−1^ and 3624 cm^−1^ are ascribed to vibrations of the inner-surface hydroxyl groups. And two apparent peaks at 585, and 635 cm^−1^ were assigned to the stretching vibration of the Fe–O groups in the Fe_3_O_4_ units. In addition to the characteristic peaks of HS and Fe_3_O_4_, the FTIR spectra of HS-Alginate /Fe_3_O_4_ (Fig. 3b) present the notable peak at 1640 cm^−1^ is owing to the C=O stretching of the alginic acid unit, as well as the peak at 2978 cm^−1^, can be attributed to the C–H stretching vibrations from the alginate backbone. In the FTIR spectrum of HS-Alginate-Ag/Fe_3_O_4_ nanocomposite, all of the typical peaks of HS, alginate, and Fe_3_O_4_ can be definitely distinguished, such as the C–O and –OH vibrations, and Si–O, Al–O–H stretching vibrations (Fig. 3c). However, several shifts are also observed. These shifts can be recognized as an indication that the hydroxyl and carboxyl groups are responsible for the Ag nanoparticle stabilization.

**Figure 4:**
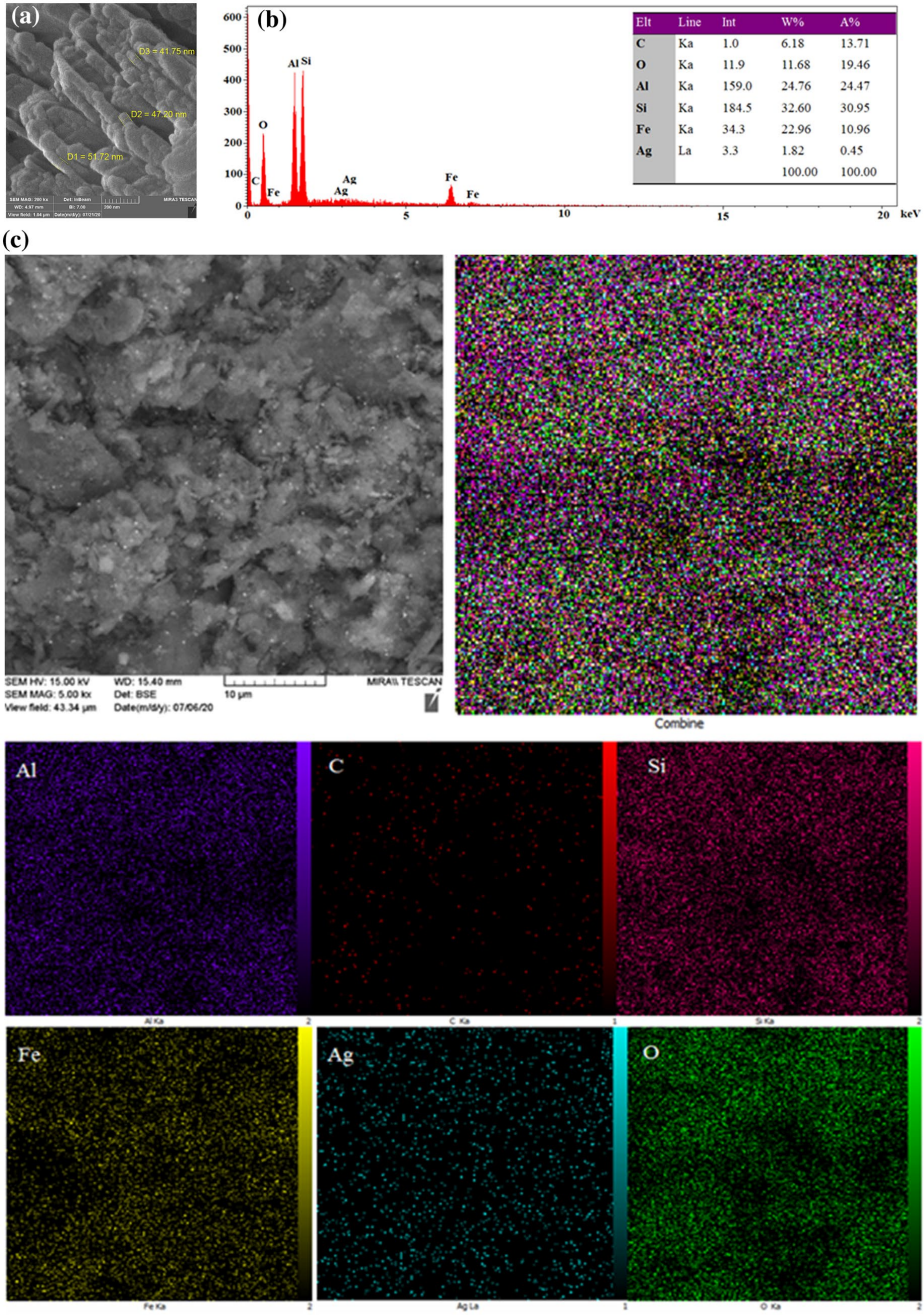
(**a**) FESEM images, (**b**) EDS, (**c**) Mapping of HS-Alginate-Ag/Fe_3_O_4_.

**Figure 5:**
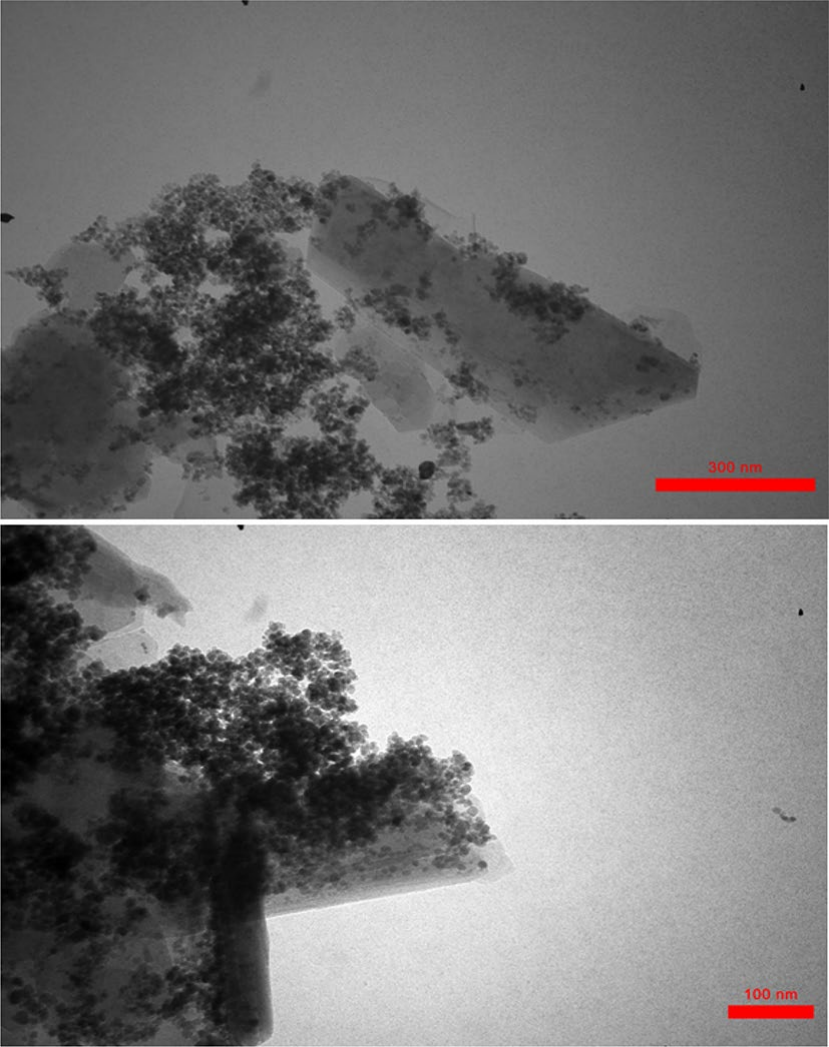
TEM images of HS-Alginate-Ag/Fe_3_O_4_.

The morphology and chemical composition of HS-Alginate-Ag/Fe_3_O_4_ were determined through FESEM, EDS, mapping, and TEM. As shown in Figs. 4 and 5 it was clear that HS has a diameter in the range of 41–52 nm, and the HS nanotubes were open-ended. In the FESEM image, it can be revealed some roughness on the surface of HS owing to grafted alginate. Also, the HS structures have a slight agglomeration. However, it showed that its surface morphology does not change very in the presence of Fe_3_O_4_ and Ag (Fig. 4a). As can be observed in Fig. 5, the HS-Alginate-Ag/Fe_3_O_4_ nanocatalyst has an almost uniform size distribution. The EDS (Fig. 4b) represented that the constituents for the HS-Alginate-Ag/Fe_3_O_4_ were Al, Si, O, Ag, C, and Fe. According to mapping (Fig. 4c), these elements were irregularly dispersed on the surface of HNTs.

The TEM image in Fig. 5 shows a defect in the HS surface that results in rough exterior walls, which is consistent with the FESEM image. The strong interaction of the polymer between the substrate (halloysite) and the nanoparticles, especially the Fe_3_O_4_ nanoparticles due to the carboxylic acid functional groups, as well as the flexibility of the polymer, has caused the nanoparticle and polymer images to be seen as mixtures that are almost far apart. This could be because polymers are also present between halloysites.

### Investigation of the catalytic activity of HS-Alginate-Ag/Fe_3_O_4_

Subsequently, hydrogenation of nitrobenzene was selected as the model reaction to investigate the catalytic activity of the HS-Alginate-Ag/Fe_3_O_4_. Therefore, the model reaction was firstly tested in the presence of (0.04 g) at 70 °C in H_2_O as solvent that was mild and environmentally benign conditions. Such conditions gave aniline in 50% yields after 1 h. To improve the yield of model reaction and decrease its time, the used solvent, the amount of HS-Alginate-Ag/Fe_3_O_4_, and the temperature were changed. It was found that running the reaction at ambient temperature gives the desired products in higher yields. By studying more temperatures, 50 °C was chosen as the optimized reaction temperature. Accordingly, by investigating the amounts of HS-Alginate-Ag/Fe_3_O_4_, it was found that the optimized amount is 0.04 g HS-Alginate-Ag/Fe_3_O_4_ and further escalation didn’t increase the product yield. Furthermore, among the tested solvents, water was the best one. Under the optimized conditions, hydrogenation of nitrobenzene gave anilines in 100% yields, respectively, after 30 min (Table 1, entry 7).
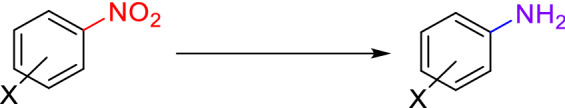

**Table 1:** Optimization conditions for reduction of nitro aromatic compounds.

Entry	Catalyst amount (g)	solvent	Temperature (°C)	Time (min)	Yield (%)	
1	0.04	CH_3_CN	50	40		10
2	0.04	CH_2_Cl_2_	Reflux	45		Trace
3	0.04	DMF	50	40		10
4	0.04	EtOH	50	30		70
5	0.04	H_2_O	Reflux	30		85
6	0.04	H_2_O	70	35		70
**7**	**0.04**	**H**_**2**_**O**	**50**	**30**		**100**
8	0.04	H_2_O	r.t	45		45
9	0.03	H_2_O	50	30		95
10	0.05	H_2_O	50	30		100

Bold indicates the optimal values of the reaction conditions

The generality of this protocol was then examined using different starting materials to produce various aromatic amines. The results confirmed that HS-Alginate-Ag/Fe_3_O_4_ can catalyze the hydrogenation reaction of all applied substrates to give the corresponding amine compounds at short reaction times and in high efficiencies (Table 2).

**Table 2:** Reduction of aromatic nitro compounds in the presence of HS-Alginate-Ag/Fe_3_O_4_ and NaBH_4_.

Entry	Product	Time (min)	Yield (%)	Melting point (Abs./ Lit.)^1^
1		25	100	143–144/145–147
2		32	99	100–103/102–104
3		30	100	187/184 (boiling point)
4		60	92	18–19/19–20
5		60	90	187/186–190
6		30	100	78/77–79
7		35	100	29–30/28–30
8		120	95	180/187–189
9	C_2_H_5_–NH_2_	40	96	20–21/19–20

### Kinetic study

The catalytic reduction of 4-nitrophenol was selected as a model reaction to appraise the catalytic activity of the HS-Alginate-Ag/Fe_3_O_4_ nanocatalysts. UV–Vis absorption spectra were used to monitor the concentration changes. The kinetics of this reaction was investigated with HS-Alginate-Ag/Fe_3_O_4_ nanocatalyst. It followed the pseudo-first-order kinetics concerning the concentration of 4-nitrophenol as follows:

$$\mathrm{ln}\frac{{C}_{t}}{{C}_{0}}= -kt$$

where C_t_ and C_0_ are the 4-nitrophenol concentrations at time t and at the beginning time, respectively, and k is the apparent rate constant. The plot of ln (C_t_ /C_0_) vs time was obtained and a good linear correlation was observed (0.99). This phenomenon also showed that this reaction followed pseudo-first-order kinetics. From the slope of this equation, the apparent rate constant (K) for the reduction of 4-nitrophenol was obtained of 0.047 s^−1^^41,42^.

### Study of reusability

**Figure 6:**
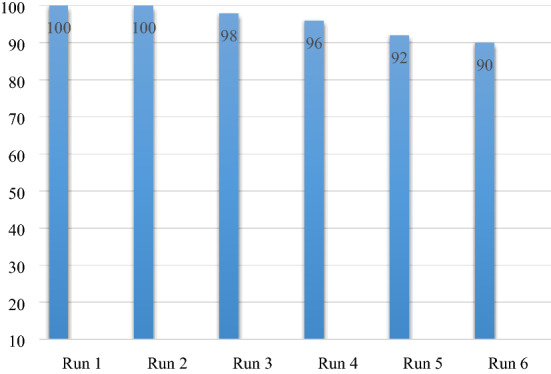
Reusability test of the HS-Alginate-Ag/Fe_3_O_4_ for model reaction.

The results obtained from the reusability evaluation of HS-Alginate-Ag/Fe_3_O_4_ in the model reaction are as follows. By completion of each cycle, the catalyst was separated, rinsed with EtOH, dried and reused in the next cycle. As shown by the results in Fig. 6, recycling the catalyst up to 6 runs showed no important loss of its catalytic activity. Since the catalyst contains magnetic Fe_3_O_4_ nanoparticles, it is easily separated from the reaction mixture by an external magnetic field. Now, after six times of recovery, observing good efficiency as well as ICP (Ag content: 1.2% to 1.1% after 6 times) results showed that the catalyst has good magnetic properties and high stability.

## Conclusions

The construction of cost-effective and reusable catalytic systems by using low-priced compounds and simple methods is an attractive research field for organic chemists. This study focused on the modification of halloysite material through three strategies including doping of alginate, deposition of silver nanoparticles, and Fe_3_O_4_ dispersion on the support surface which was synthesized for the first time. The as-prepared HS-Alginate-Ag/Fe_3_O_4_ was utilized as a suitable and recoverable nanocatalyst for the reduction of nitro aromatic compounds to the related amine compounds. The obtained HS-Alginate-Ag/Fe_3_O_4_ nanocatalyst presented a good performance in the reduction of nitro aromatic compounds to the amine aromatic compounds for a broad range of materials under moderate conditions. Recycling experiment of HS-Alginate-Ag/Fe_3_O_4_ was performed using water as a green solvent and NaBH_4_ as a hydrogen donor. This heterogeneous HS-Alginate-Ag/Fe_3_O_4_ nanocatalyst exhibited acceptable stability and recovered by a magnet and reutilized several times with a low decrease in its efficiency.

## References

[1] Gholinejad M, Zareh F, Nájera C (2018). Nitro group reduction and Suzuki reaction catalysed by palladium supported on magnetic nanoparticles modified with carbon quantum dots generated from glycerol and urea. Appl. Organomet. Chem..

[2] Ma X, Zhou YX, Liu H, Li Y, Jiang HL (2016). A MOF-derived Co–CoO@ N-doped porous carbon for efficient tandem catalysis: Dehydrogenation of ammonia borane and hydrogenation of nitro compounds. Chem. Commun..

[3] Rahman A, Jonnalagadda SB (2008). Swift and selective reduction of nitroaromatics to aromatic amines with Ni–boride–silica catalysts system at low temperature. Catal. Lett..

[4] Sadjadi S, Lazzara G, Heravi MM, Cavallaro G (2019). Pd supported on magnetic carbon coated halloysite as hydrogenation catalyst: Study of the contribution of carbon layer and magnetization to the catalytic activity. Appl. Clay Sci..

[5] Hernández-Gordillo A, González VR (2015). Silver nanoparticles loaded on Cu-doped TiO2 for the effective reduction of nitro-aromatic contaminants. Chem. Eng. J..

[6] Wu F, Qiu LG, Ke F, Jiang X (2013). Copper nanoparticles embedded in metal–organic framework MIL-101 (Cr) as a high performance catalyst for reduction of aromatic nitro compounds. Inorg. Chem. Commun..

[7] Nandanwar SU, Chakraborty M (2012). Synthesis of colloidal CuO/γ-Al2O3 by microemulsion and its catalytic reduction of aromatic nitro compounds. Chin. J. Catal..

[8] Kalbasi RJ, Nourbakhsh AA, Babaknezhad F (2011). Synthesis and characterization of Ni nanoparticles-polyvinylamine/SBA-15 catalyst for simple reduction of aromatic nitro compounds. Catal. Commun..

[9] Cui X, Liang K, Tian M, Zhu Y, Ma J, Dong Z (2017). Cobalt nanoparticles supported on N-doped mesoporous carbon as a highly efficient catalyst for the synthesis of aromatic amines. J. Colloid Interface Sci..

[10] Shukla A, Singha RK, Sasaki T, Bal R (2015). Nanocrystalline Pt-CeO 2 as an efficient catalyst for a room temperature selective reduction of nitroarenes. Green Chem..

[11] Aditya T, Pal A, Pal T (2015). Nitroarene reduction: A trusted model reaction to test nanoparticle catalysts. Chem. Commun..

[12] Mei Y, Sharma G, Lu Y, Ballauff M, Drechsler M, Irrgang T, Kempe R (2005). High catalytic activity of platinum nanoparticles immobilized on spherical polyelectrolyte brushes. Langmuir.

[13] Dotzauer DM, Bhattacharjee S, Wen Y, Bruening ML (2009). Nanoparticle-containing membranes for the catalytic reduction of nitroaromatic compounds. Langmuir.

[14] Kuroda K, Ishida T, Haruta M (2009). Reduction of 4-nitrophenol to 4-aminophenol over Au nanoparticles deposited on PMMA. J. Mol. Catal. A.

[15] Sravanthi K, Ayodhya D, Swamy PY (2019). Green synthesis, characterization and catalytic activity of 4-nitrophenol reduction and formation of benzimidazoles using bentonite supported zero valent iron nanoparticles. Mater. Sci. Energy Technol..

[16] Qiu X, Liu Q, Song M, Huang C (2016). Hydrogenation of nitroarenes into aromatic amines over Ag@ BCN colloidal catalysts. J. Colloid Interface Sci..

[17] Rostami-Vartooni A, Nasrollahzadeh M, Alizadeh M (2016). Green synthesis of seashell supported silver nanoparticles using Bunium persicum seeds extract: Application of the particles for catalytic reduction of organic dyes. J. Colloid Interface Sci..

[18] Shiraishi Y, Toshima N (2000). Oxidation of ethylene catalyzed by colloidal dispersions of poly (sodium acrylate)-protected silver nanoclusters. Colloids Surf. A.

[19] Yan H, Zhao L, Shang W, Liu Z, Xie W, Qiang C, Xiong Z, Zhang R, Li B, Sun X, Kang F (2017). General synthesis of high-performing magneto-conjugated polymer core–shell nanoparticles for multifunctional theranostics. Nano Res..

[20] Zhang J, Zhang Y, Chen Y, Du L, Zhang B, Zhang H, Liu J, Wang K (2012). Preparation and characterization of novel polyethersulfone hybrid ultrafiltration membranes bending with modified halloysite nanotubes loaded with silver nanoparticles. Ind. Eng. Chem. Res..

[21] Lisuzzo L, Cavallaro G, Milioto S, Lazzara G (2019). Layered composite based on halloysite and natural polymers: A carrier for the pH controlled release of drugs. New J. Chem..

[22] Pan J, Yao H, Xu L, Ou H, Huo P, Li X, Yan Y (2011). Selective recognition of 2, 4, 6-trichlorophenol by molecularly imprinted polymers based on magnetic halloysite nanotubes composites. J. Phys. Chem. C.

[23] Bates TF, Hildebrand FA, Swineford A (1950). Morphology and structure of endellite and halloysite. Am. Miner..

[24] Lvov Y, Wang W, Zhang L, Fakhrullin R (2016). Halloysite clay nanotubes for loading and sustained release of functional compounds. Adv. Mater..

[25] Lvov Y, Abdullayev E (2013). Functional polymer–clay nanotube composites with sustained release of chemical agents. Prog. Polym. Sci..

[26] Liu M, Jia Z, Jia D, Zhou C (2014). Recent advance in research on halloysite nanotubes-polymer nanocomposite. Prog. Polym. Sci..

[27] Zeng G, He Y, Ye Z, Yang X, Chen X, Ma J, Li F (2017). Novel halloysite nanotubes intercalated graphene oxide based composite membranes for multifunctional applications: Oil/water separation and dyes removal. Ind. Eng. Chem. Res..

[28] Hebbar RS, Isloor AM, Ananda K, Ismail AF (2016). Fabrication of polydopamine functionalized halloysite nanotube/polyetherimide membranes for heavy metal removal. J. Mater. Chem. A.

[29] Ghanbari M, Emadzadeh D, Lau WJ, Lai SO, Matsuura T, Ismail AF (2015). Synthesis and characterization of novel thin film nanocomposite (TFN) membranes embedded with halloysite nanotubes (HNTs) for water desalination. Desalination.

[30] Hashemifard SA, Ismail AF, Matsuura T (2011). Mixed matrix membrane incorporated with large pore size halloysite nanotubes (HNT) as filler for gas separation: Experimental. J. Colloid Interface Sci..

[31] Zhang Y, He X, Ouyang J, Yang H (2013). Palladium nanoparticles deposited on silanized halloysite nanotubes: Synthesis, characterization and enhanced catalytic property. Sci. Rep..

[32] Li J, Zhou M, Ye Z, Wang H, Ma C, Huo P, Yan Y (2015). Enhanced photocatalytic activity of gC 3 N 4–ZnO/HNT composite heterostructure photocatalysts for degradation of tetracycline under visible light irradiation. RSC Adv..

[33] Li C, Wang J, Feng S, Yang Z, Ding S (2013). Low-temperature synthesis of heterogeneous crystalline TiO 2–halloysite nanotubes and their visible light photocatalytic activity. J. Mater. Chem. A.

[34] Gómez L, Hueso JL, Ortega-Liébana MC, Santamaría J, Cronin SB (2014). Evaluation of gold-decorated halloysite nanotubes as plasmonic photocatalysts. Catal. Commun..

[35] Schexnailder P, Schmidt G (2009). Nanocomposite polymer hydrogels. Colloid Polym. Sci..

[36] Vieira EF, Cestari AR, Airoldi C, Loh W (2008). Polysaccharide-based hydrogels: Preparation, characterization, and drug interaction behaviour. Biomacromol.

[37] Boanini E, Rubini K, Panzavolta S, Bigi A (2010). Chemico-physical characterization of gelatin films modified with oxidized alginate. Acta Biomater..

[38] Zhao P, Li X, Wang Y, Yan L, Guo L, Huang L, Gao W (2020). Characterisation and saccharide mapping of polysaccharides from four common Polygonatum spp. Carbohydr. Polym..

[39] Paques JP, van der Linden E, van Rijn CJ, Sagis LM (2014). Preparation methods of alginate nanoparticles. Adv. Colloid Interface. Sci..

[40] Kumar KS, Kumar VB, Paik P (2013). Recent advancement in functional core-shell nanoparticles of polymers: Synthesis, physical properties, and applications in medical biotechnology. J. Nanopart..

[41] Guan H, Chao C, Lu Y, Shang H, Zhao Y, Yuan S, Zhang B (2016). PtNi nanoparticles embedded in porous silica microspheres as highly active catalysts for p-nitrophenol hydrogenation to p-aminophenol. J. Chem. Sci..

[42] Shang H, Du L, Guan H, Zhang B, Xiang X (2018). Ternary composite of biomass porous Carbon/SnO2/Pt: An efficient catalyst for reduction of aromatic nitro compounds. ChemistrySelect.

